# The comparison of percutaneous kyphoplasty and vertebroplasty for the management of stage III Kummell disease without neurological symptoms

**DOI:** 10.1186/s12893-022-01770-1

**Published:** 2022-08-20

**Authors:** Hanwen Li, Yingchuang Tang, Zixiang Liu, Huilin Yang, Zhigang Zhang, Kai Zhang, Kangwu Chen

**Affiliations:** Department of Orthopedic Surgery, The First Affiliated Hospital of Soochow University, Suzhou, 215006 Jiangsu China

**Keywords:** Kummell’s disease, PVP, PKP, OVCF

## Abstract

**Purpose:**

To compare the clinical and radiological outcomes of percutaneous kyphoplasty (PKP) and percutaneous vertebroplasty (PVP) in the treatment of stage III Kummell disease without neurological deficit.

**Methods:**

This retrospective study involved 41 patients with stage III Kummell disease without neurological deficit who underwent PKP or PVP from January 2018 to December 2019. Demographic data and clinical characteristics were comparable between these two groups before surgery. Operation time, volume of injected bone cement, intraoperative blood loss and time of hospital stay were analyzed. Visual analog scale (VAS) scoring and Oswestry disability index (ODI) scoring were assessed for each patient before and after operation. Radiographic follow-up was assessed by the height of anterior (Ha), the height of middle (Hm), Cobb’s angle, and Vertebral wedge ratio (VWR). The preoperative and postoperative recovery values of these data were used for comparison.

**Results:**

The two groups showed no significant difference in demographic features (p > 0.05). What’s more, the operation time, intraoperative blood loss, and time of hospital stay revealed no sharp statistical distinctions either (p > 0.05), except PKP used more bone cement than PVP (7.4 ± 1.7 mL vs 4.7 ± 1.4 mL, p < 0.05). Radiographic data, such as the Ha improvement ratio (35.1 ± 10.2% vs 16.2 ± 9.4%), the Hm improvement ratio (41.8 ± 11.3% vs 22.4 ± 9.0%), the Cobb’s angle improvement (10.0 ± 4.3° vs 3.5 ± 2.1°) and the VWR improvement ratio (30.0 ± 10.6% vs 12.7 ± 12.0%), were all better in PKP group than that in PVP group (p < 0.05). There were no statistical differences in the improvement of VAS and ODI 1-day after the surgery between these two groups (p > 0.05). However, at the final follow-up, VAS and ODI in PKP group were better than that in PVP (p < 0.05). Cement leakage, one of the most common complications, was less common in the PKP group than that in the PVP group (14.3% vs 45.0%, p < 0.05). And there was 1 case of adjacent vertebral fractures in both PKP and PVP (4.8% vs 5.0%, p > 0.05), which showed no statistical difference, and there were no severe complications recorded.

**Conclusions:**

For stage III Kummell disease, both PKP and PVP can relieve pain effectively. Moreover, PKP can obtain more satisfactory reduction effects and less cement leakage than PVP. We suggested that PKP was more suitable for stage III Kummell disease without neurological deficit compared to PVP from a vertebral reduction point of view.

## Introduction

Kummell's disease, first reported by Kummell in 1895 [1], was a delayed complication of osteoporotic vertebral compression fracture (OVCF). The main characteristic is that patients with a slight trauma tend to develop a symptomatic and progressive angular kyphosis after a short asymptomatic period. It is also known as nonunion after OVCF or delayed vertebral osteonecrosis after trauma [2]. Kummell's disease was divided into three stages based on different clinical symptoms, radiographs, and the magnetic resonance imaging [3]. To be specific, the vertebral body height loss is less than 20% in stage I of Kummell disease, with or without adjacent intervertebral disc degeneration; in stage II the vertebral body height loss is greater than20% and is always along with adjacent intervertebral disc degeneration. Stage III Kummell disease is characterized by posterior breakage with or without spinal cord compression. Conservative treatment was once performed for Kummell's disease but it is often ineffective [4]. In previous studies, PVP and PKP were reported to achieve a good effect in the treatment of stage I and II Kummell disease [5, 6]. But for the stage III Kummell disease, the treatment remains controversial [7].

The traditional surgery treatment aimed to correct kyphosis, achieve decompression, fixation, and fusion, but it has more destruction of paravertebral muscles and ligament and more blood loss [8]. What’s worse, to those who have severe osteoporosis, internal fixation has a high probability of failure [8]. PKP or PVP may be the candidate treatment for stage III Kummell disease. In our study, we retrospectively analyzed and compared the safety and efficacy of the PVP and PKP for treating patients who suffered from stage III Kummell disease without nerve injury.

## Materials and methods

### Inclusion and exclusion criteria

This is a retrospective study.

*Inclusion criteria* ①Bending on clinical symptoms and imaging examination, patients who met the reported diagnostic criteria [3] for stage III Kummell disease; ②Bone density T value < − 2.5 on dual‑energy X‑ray absorptiometry (DXA), which is in accordance with the diagnostic criteria for osteoporosis; ③Magnetic resonance imaging (MRI) showed no spinal canal involvement and no nerve injury, which is consistent with the symptoms; ④Only one single responsible vertebra is involved.

*Exclusion criteria* ①Patients with pathological vertebral fracture, serious internal medical diseases, like spinal metastatic tumor, vertebral tuberculosis. ②Patients with severe cardiopulmonary, liver and kidney dysfunction who cannot tolerate surgery. ③Patients with incomplete clinical data.

### Patients

41 patients with stage III Kummell disease without neurologic deficits who underwent PKP or PVP between January 2018 and December 2019 were recruited. Demographic data for the 2 groups are presented in Table 1.

**Table 1:** Demographic data of patients

Variable	PKP Group	PVP Group	P Value
Number of patients	21	20	
Age, mean ± SD	70.7 ± 7.3	70.0 ± 7.4	0.758
Sex, number			
Male	6	5	0.796
Female	15	15	
Medical history (months), mean ± SD	5.6 ± 2.9	5.6 ± 2.8	0.981
BMD, mean ± SD	− 3.5 ± 0.4	− 3.3 ± 0.5	0.386
Responsible segment, number			
T10	1	1	0.951
T11	0	1	
T12	2	2	
L1	11	9	
L2	4	5	
L3	2	1	
L4	1	1	
Follow-up (months), mean ± SD	37.7 ± 7.7	39.6 ± 5.1	0.355

All patients received conservative treatment for at least 3 months before admission; thus, we did not diagnose Kummell’s disease before 3 months [9]. The patients were informed of the advantages and disadvantages of PKP and PVP before they made the choice. Meanwhile, they were instructed that there was no sufficient evidence-based medicine showing which one was better.

### Surgical information

For PKP group, patients were performed in a prone, lordotic posture to maintain posterior extension of the spine under general anesthesia. The standard procedure for PKP surgery can be referred to our previously published article [10]. In short, after disinfection, bilateral transpedicular puncture was performed, bilateral balloons were placed under the endplate through the working tunnel. Balloons were inflated gently to restore the height of the affected vertebra and deflated after elevating the superior endplate. After the balloon was removed, polymethylmethacrylate cement was used to fill the pre-formed hollow. The whole process was monitored by C-arm fluoroscopy.

For PVP groups, most of the steps are similar, but without the balloon. After a working tunnel was established, polymethylmethacrylate bone cement was pushed into the vertebra directly.

### Clinical and radiologic assessment

The visual analogue scale (VAS score 0–10; 0 no pain at all; 10 the worst imaginable) system was employed to evaluate back pain control. Impact on the patient’s daily life was assessed using the Oswestry Disability Index (ODI) questionnaire [11, 12]. Radiographs were taken to measure the rate of cement leakages and refracture, the anterior, middle and posterior vertebral heights, Cobb’s angle and Vertebral wedge ratio [11] of the fractured vertebral body before and after surgery. The operation time, amounts of cement injected, time of hospital stay and intraoperative blood loss of the two procedures were recorded.

All radiographic measurements were performed in a double-blinded fashion by 2 orthopedic surgeons.

### Statistical analysis

SPSS 21.0 software was applied to carry out all analyzes. Data was calculated as mean ± standard deviation. Preoperative and postoperative measurement data were assessed by using paired t-test and χ^2^ test. A P value < 0.05 was considered statistically significant.

## Results

There were no significant differences between groups in terms of preoperative demographic data (Table 1). All patients tolerated the operation well. For average operation time, PKP group was 52.6 ± 15.9 min and PVP group was 46.3 ± 12.8 min. Blood loss during the operation was minimal, 18.1 ± 4.6 mL in PKP group and 17.6 ± 3.7 mL in PVP group. The average volume of cement injected in PKP and PVP group was 7.4 ± 1.7 mL and 4.7 ± 1.4 mL, respectively (Table 2).

**Table 2:** Comparison of operation time, intraoperative blood loss, hospitalization stays, and volume of cement injected

Variable	PKP Group	PVP Group	P value
Operation time (min)	52.6 ± 15.9	46.3 ± 12.8	0.167
Intraoperative blood loss (mL)	18.1 ± 4.6	17.6 ± 3.7	0.707
Hospitalization stays (days)	3.5 ± 0.7	3.6 ± 0.6	0.716
Cement volume (mL)	7.4 ± 1.7	4.7 ± 1.4	< 0.001

Patients had follow-up from 24 to 48 months. There were significant improvements in both groups (p < 0.05) in the VAS, ODI score at the 1‑day postoperatively and at the final follow-up compared with the preoperative values (Table 3). In PKP group, the VAS pain score decreased from a preoperative value of 8.1 ± 0.9 to a postoperative value of 2.4 ± 0.5 (p < 0.05), and further 2.5 ± 0.5 at final follow-up. In PVP group, this score also decreased from a preoperative value of 8.2 ± 0.8 to a postoperative value of 2.3 ± 0.7 (p < 0.05), and finally 3.1 ± 0.7. There was no significant difference in VAS score at the 1‑day postoperatively between the PVP group and PKP group (p > 0.05). However, at the final follow-up, PKP group turned out to be a better procedure (p < 0.05). As for the ODI score, the same trend was observed.

**Table 3:** Mean improvement in VAS and ODI

Variable	PKP group	PVP group	P value
VAS-preoperative	8.1 ± 0.9	8.2 ± 0.8	0.978
VAS-postoperative	2.4 ± 0.5^a^	2.3 ± 0.7^a^	0.658
VAS-final follow-up	2.5 ± 0.5^a,b^	3.1 ± 0.7^a^^,^^c^	0.004
ODI (%)-preoperative	80.2 ± 6.8	81.4 ± 5.7	0.540
ODI (%)-postoperative	25.5 ± 3.5^a^	25.5 ± 2.9^a^	0.979
ODI (%)-final follow-up	25.8 ± 2.9^a,b^	31.7 ± 3.9^a,c^	< 0.001

^a^P < 0.05 versus preoperative values

^b^P > 0.05 versus postoperative values

^c^P < 0.05 versus postoperative values

**Fig. 1:**
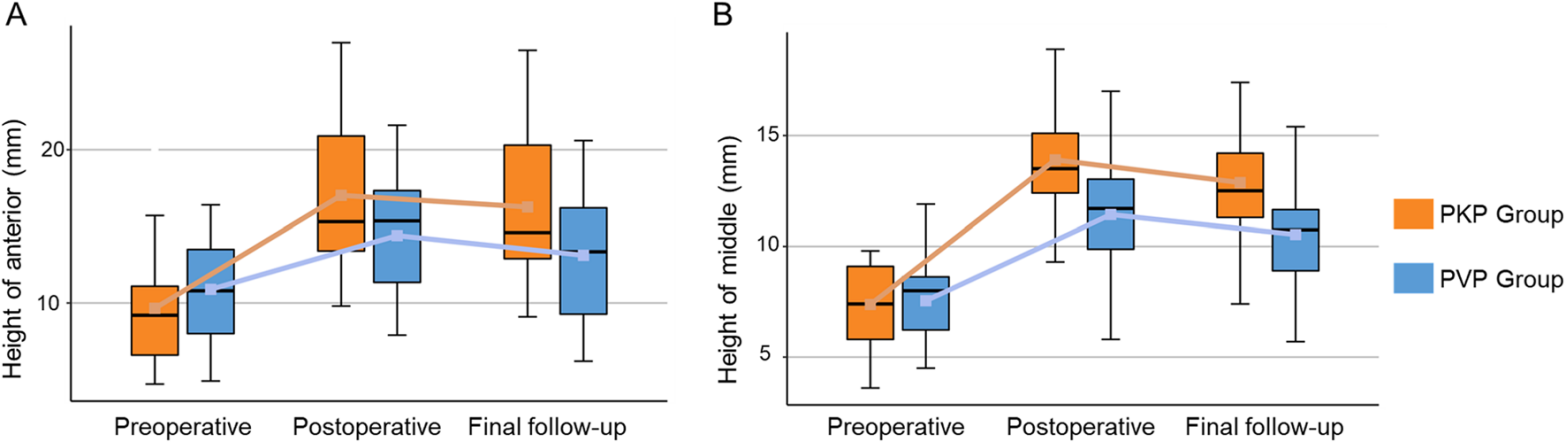
Box plots show the summary of baseline, follow-up, and changes by group. The horizontal lines in the boxplots from bottom to top show the 25th, 50th (median), and 75th percentiles. The dot in the boxplot indicates the mean. The whiskers indicate the highest and lowest values no further than 1.5 times the interquartile range. **A** Changes in the height of anterior. **B** Changes in the height of middle

Significant increases of the anterior and middle vertebral heights were observed after surgery too (p < 0.05). However, the change of the posterior was not significant (p = 0.273). The improvements of PKP and PVP group in Cobb’s angle were 10.0 ± 4.3° and 3.5 ± 2.1°, and in VWR were 30.0 ± 10.6% and 12.7 ± 12.0%, respectively (Table 4). PKP displayed better recovery capability than PVP (p < 0.05). What’s more, the correction was almost maintained at the final follow-up (Fig. 1). Asymptomatic cement leakage occurred with 9 cases in PVP group and 3 cases in PKP group (Table 4), the probability of bone cement leakage was lower in the PKP group (p < 0.05). Adjacent vertebral fractures occurred in 1 case of the PKP group and 1 case of the PVP group. There was not significant difference in the number of adjacent vertebral fractures between the two groups (p > 0.05). There was no other serious complication.

**Table 4:** Clinical and radiographic data

Variable	PKP group	PVP group	P value
Vertebral body height ratios improvement (%)^a^			
Anterior	35.1 ± 10.2	16.2 ± 9.4	< 0.001
Middle	41.8 ± 11.3	22.4 ± 9.0	< 0.001
Posterior	8.0 ± 8.6	5.6 ± 4.7	0.273
Cobb’s angle improvement	10.0 ± 4.3	3.5 ± 2.1	< 0.001
VWR improvement (%)^b^	30.0 ± 10.6	12.7 ± 12.0	< 0.001
Cement leakage, number	3	9	0.025
Adjacent vertebral fractures, number	1	1	0.972

^a^Vertebral body height ratio (%) = (fractured vertebral body height/normal vertebral body height) × 100%

^b^VWR, Vertebral wedge ratio (%) = (fractured vertebral body anterior height/fractured vertebral body posterior height) × 100%

## Illustrative case

**Fig. 2:**
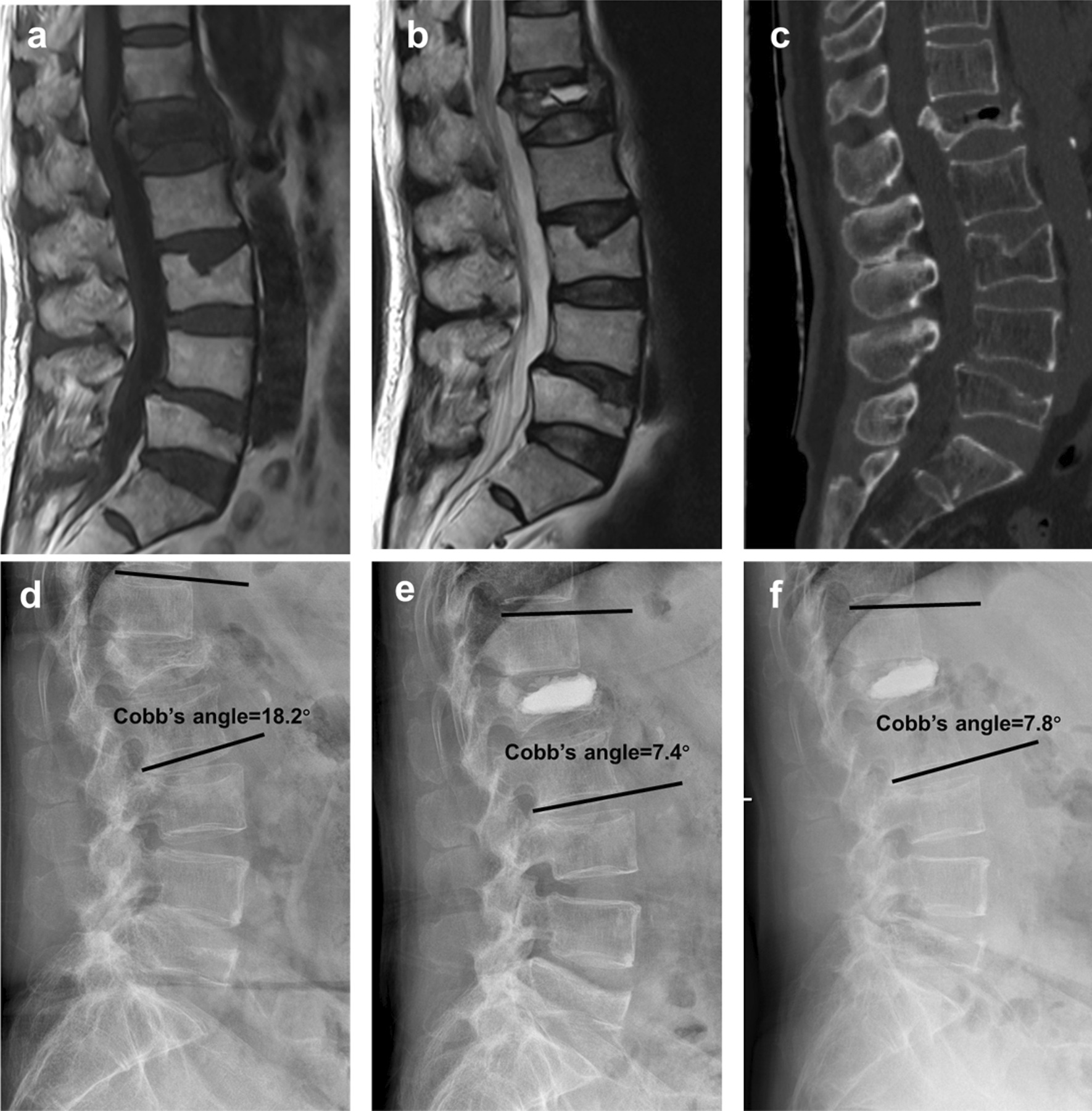
A 64-year-old woman who had L1 stage III Kummell disease without neurological symptom was treated with PKP. **a**–**c** The preoperative MRI T1WI, MRI T2WI and CT films showed a chronic osteoporotic vertebral compressive fracture. **d–f** The preoperative, postoperative and final follow-up X-ray films displayed the vertebral height and Cobb’s angle was well recovered

**Fig. 3:**
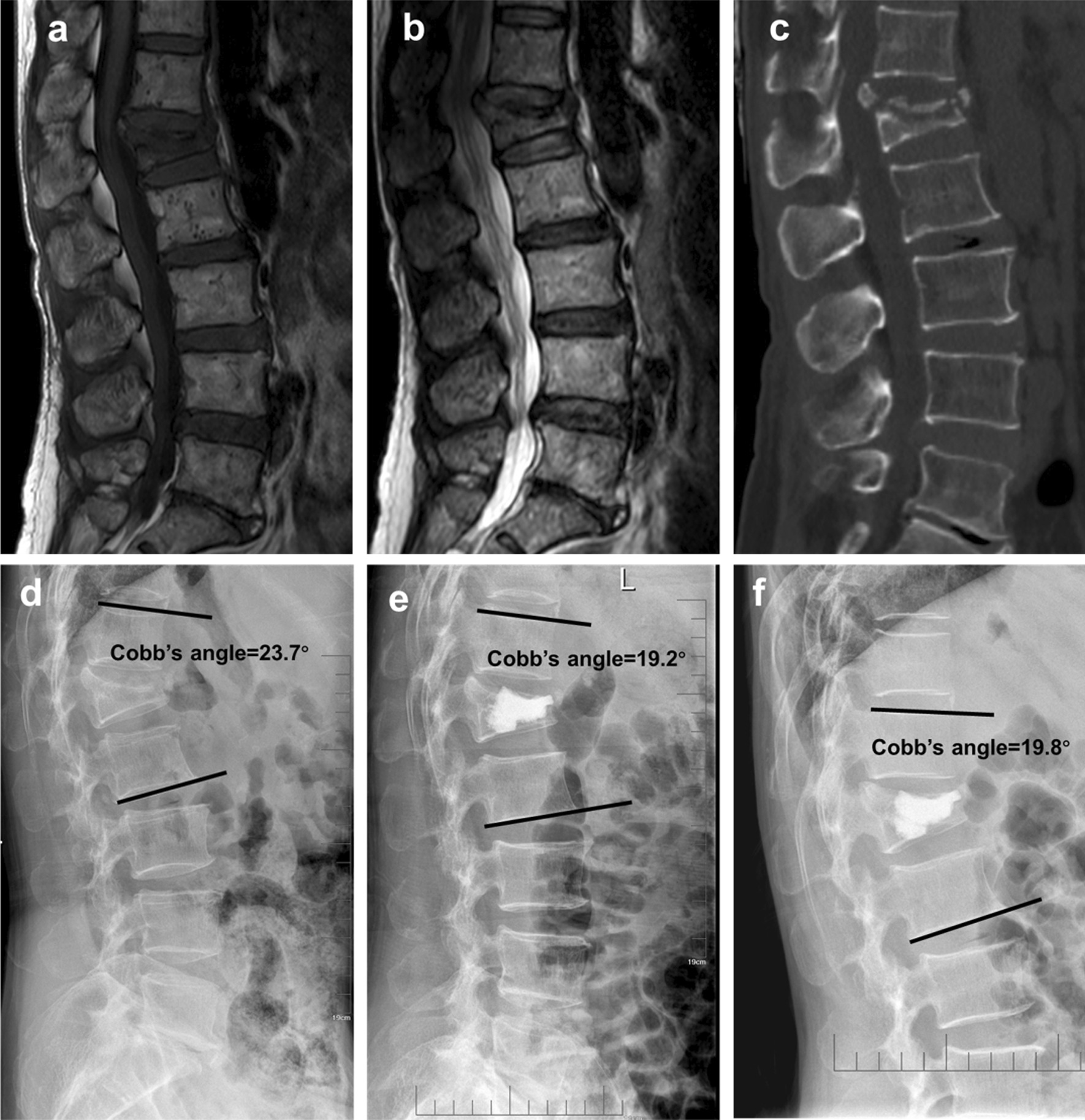
A 63-year-old woman who had L1 stage III Kummell disease without neurological symptom was treated with PVP. **a–c** The preoperative MRI T1WI, MRI T2WI and CT films showed a chronic osteoporotic vertebral compressive fracture. **d–f** The preoperative, postoperative and final follow-up X-ray films displayed the vertebral height and Cobb’s angle was recovered a little

Vertebral body height and local kyphotic angle showed significant recovery after PKP surgery (Fig. 2), PVP can also restore vertebral height, but not as good as PKP (Fig. 3). Both PKP and PVP will lose a little vertebral height during the follow-up.

## Discussion

Kummell’s disease is characterized by delayed osteoporotic vertebral collapse and chronic back pain [9, 13]. Conservative treatment tends to be ineffective [14], patients with stage III Kummell disease often have severe osteoporosis and it is a huge risk for them to undergo open surgery [15–17]. Therefore, open surgery may not be the first choice for these patients. Given the possibility of technical difficulty and cement leakage [18], PKP and PVP are still cautious for severe OVCF treatment. The treatment for stage III Kummell disease without neurological deficits remains controversial.

Studies have shown that both PKP and PVP can relieve the chronic pain and correct the kyphosis in stage I and II Kummell disease [19, 20]. Similar phenomenon was observed in our study, we found that PKP and PVP both could effectively alleviate patients’ back pain in stage III Kummell disease. The VAS score and ODI score was significantly improved after surgery in both groups. However, there was no significant difference in VAS or ODI score between the two groups at one day after surgery, but VAS and ODI score in PKP group was significantly lower than that in PVP group at final follow-up. This may be due to the Cobb’s angle correction. As we know, kyphosis tends to cause chronic pain [21]. In our study, we found that Ha, Hm improvement ratio and Cobb’s angle, VWR improvement in PKP group were obviously better than that in PVP group. Moreover, we also discovered that the average volume of bone cement in PKP group was more than that in PVP group. The possible reason may be that bone cement is usually confined to the vertebral fissure in PVP treatment, while in PKP treatment, bone cement can maintain the correction of hyperextension kyphosis with the help of an expanded balloon. In a word, both PKP and PVP can effectively treat stage III Kummel disease and PKP can achieve better vertebral height restoration and kyphosis correction than PVP.

The most common complications of PKP and PVP are bone cement leakage and adjacent vertebral fractures [22, 23]. Wang et al. reported that compared with vertebroplasty, kyphoplasty significantly decreased the risk of cement leakage through a meta-analysis and systematic review [24]. Chang et al. found that for the treatment of Kummell’s disease, PKP has a lower rate of bone cement leakage than PVP (10.7% vs 17.2%) [25]. In our study, PKP showed a better way to avoid cement leakage than PVP (14.3%vs 45.0%), and none of these patients had obvious symptoms. The reason was mainly related to the fact that the PKP group could squeeze the surrounding cancellous bone during balloon expansion and reduce the bone cement leakage. There was 1 case of adjacent vertebral fractures in both PKP and PVP (4.8% vs 5.0%), which showed no statistical difference. This may be due to improved postoperative rehabilitation and anti-osteoporosis treatment, but also related to the small sample size. To sum up, PKP and PVP are both effective quality methods, but PKP is superior in terms of cement leakage.

According to our experience, paying enough attention before and after surgery can greatly reduce the complications of surgery. Although severe vertebral collapse and incomplete posterior wall of the vertebral body is present in stage III Kummell disease, thorough preoperative imaging examination, such as MRI and CT coronal and sagittal reconstruction, is of great importance for us to decide whether PVP or PKP can be used. Intraoperatively, precise puncture technique, moderate balloon dilation and ideal balloon placement are critical. In our experience, the ideal location of the balloon is in the anterior 3/4 of the vertebral body. What’s more, appropriate bone cement injection is helpful to reduce the leakage rate of bone cement. For patients with anterior wall defect indicated by preoperative imaging, we used graded infusion of bone cement. A small amount of bone cement is injected to firstly seal the rupture, thus improving vertebral stability. Postoperatively, patients need to receive systematic and personalized anti-osteoporosis therapy, which is important for the prevention and treatment of complications.

However, this study also had some limitations. The number of intraoperative fluoroscopy and radiation dose were not counted in this study, which will be improved in our future study. The sample size of this study is still small, and a large sample randomized controlled study is needed.

## Conclusion

To conclude, both PVP and PKP are effective in pain relief for stage III Kummell disease without neurological deficit, they have the advantages of small trauma, short operation time, and quick recovery. Compared with PVP, PKP could achieve better vertebral height restoration and kyphosis correction. Furthermore, PKP exists less cement leakage than PVP.

## Data Availability

The data that support the findings of this study are included in this manuscript, and the original files are available from the corresponding author upon reasonable request.
